# Mutational analysis of a Chinese family with oculocutaneous albinism type 2

**DOI:** 10.18632/oncotarget.19697

**Published:** 2017-07-31

**Authors:** Xiong Wang, Yaowu Zhu, Na Shen, Jing Peng, Chunyu Wang, Haiyi Liu, Yanjun Lu

**Affiliations:** ^1^ Department of Laboratory Medicine, Tongji Hospital, Tongji Medical College, Huazhong University of Science and Technology, Wuhan 430030, China; ^2^ Department of Obstetrics and Gynecology, Tongji Hospital, Tongji Medical College, Huazhong University of Science and Technology, Wuhan 430030, China

**Keywords:** OCA2, SLC45A2, mutation, splice site

## Abstract

Oculocutaneous albinism (OCA) is an autosomal recessive disorder characterized by hypopigmentation of the skin, hair, and eyes accompanied with ophthalmologic abnormalities. Molecular genetic test can confirm the diagnosis of the four subtypes of OCA (OCA1-4). Herein, we report a Chinese family with two patients affected by OCA. Mutations of *TYR*, *OCA2, TYRP1*, and *SLC45A2* were examined by using PCR-sequencing. Large deletions or duplications of *TYR* and *OCA2* were examined by Multiplex Ligation-dependent Probe Amplification (MLPA) assay. Compound heterozygous mutations of *OCA2*, (c.808-3C>G and c.2080-2A>G), were identified in both patients characterized with yellow hair and milky skin, heterochromia iridis, and nystagmus. Several computer-assisted approaches predicted that c.808-3C>G and c.2080-2A>G in *OCA2* might potentially be pathogenic splicing mutations. No exon rearrangement (deletion/duplication) of *TYR* and *OCA2* was observed in the patients by MLPA analysis. This study suggests that compound heterozygous mutations, (c.808-3C>G and c.2080-2A>G), in *OCA2* may be responsible for partial clinical manifestations of OCA.

## INTRODUCTION

Oculocutaneous albinism (OCA) is a heterogeneous and autosomal recessive disorder with an estimated prevalence of 1/17,000 worldwide, and the carrier rate is approximately 1 in 70. OCA is characterized by a reduction or complete loss of pigment in the skin, hair, and eyes accompanied by photophobia, nystagmus, strabismus, and reduced visual acuity due to melanin biosynthesis deficiency [1, 2]. OCA is broadly classified as non-syndromic and syndromic OCA based on the presence of other symptoms such as immunodeficiency, bleeding diathesis, or neurological dysfunction [3, 4]. Non-syndromic OCA includes four types, OCA1-4, and the clinical diagnosis of OCA subtype is difficult because of its variable clinical phenotype. Emerging evidence shows that molecular and genetic analyses can provide accurate diagnosis and genetic counselling.

The prevalence of different OCA subtypes significantly differs in different ethnic populations. OCA1 and OCA2 are the two most frequent types of OCA, making up 50% and 30% of all OCA cases worldwide, respectively [1, 5]. OCA1 is caused by mutations of *TYR*. OCA2-4, which are somewhat milder, are caused by mutations in *OCA2*, *TYRP1*, and *SLC45A2*, respectively. OCA2 is mainly found in Africa, and the frequencies of OCA3 and OCA4 are approximately 3% and 17% worldwide, respectively [6–8].

OCA2 is characterized by yellow, brown, or golden hair at birth. To date, 154 *OCA2* mutations have been identified, including 4 nonsense mutations, 92 missense mutations, 17 splicing mutations, 1 regulatory mutation, 20 small deletions, 7 small insertions, 2 small indels, 9 gross deletions, and 2 gross insertions/duplications. Wei *et al.* reported that 8 of 52 Chinese families including patients with OCA were identified with mutations in *OCA2*, accounting for 15.4% [9].

Genetic tests were carried out to provide an accurate genetic diagnosis and genetic counselling for a Chinese family with two patients affected by OCA characterized by yellow hair, milky skin, photophobia, nystagmus, and reduced visual acuity. Two compound heterozygous mutations (c.808-3C>G and c.2080-2A>G) in *OCA2* were identified, which may result in pathogenic splice site mutation and may be responsible for some clinical manifestations of OCA.

## RESULTS

### Clinical phenotype

**Figure 1:**
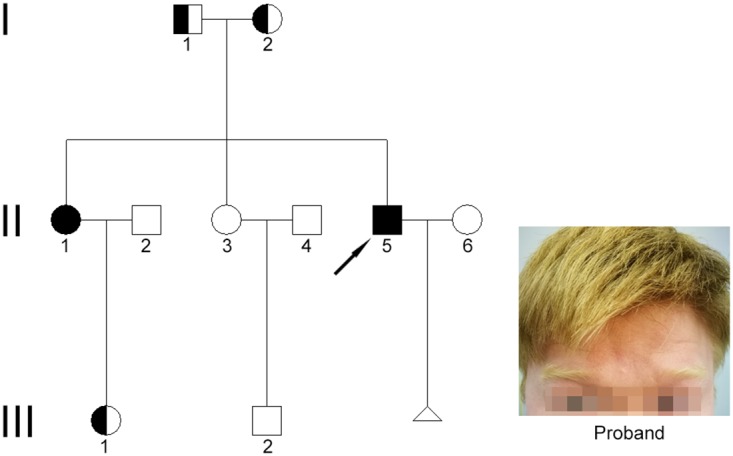
Clinical features and family pedigree A simplified pedigree is shown. Arrow indicates the proband. Square symbols denote males, and the circles denote females. A filled square indicates affected male, and filled circle indicates affected female. Half-filled square and circle indicate carriers. Triangle denotes pregnancy. Image of the hair and eyebrow of the proband is shown.

The pedigree chart and the clinical features of the male patient (proband) affected by OCA are shown in Figure 1. Both the patients have yellow eyebrows and hair, milky skin, and heterochromia iridis, accompanied with photophobia, impaired visual acuity, and nystagmus. On the other hand, unaffected family members present normal pigmentation.

### Mutation identification and analysis of *OCA2*

**Figure 2:**
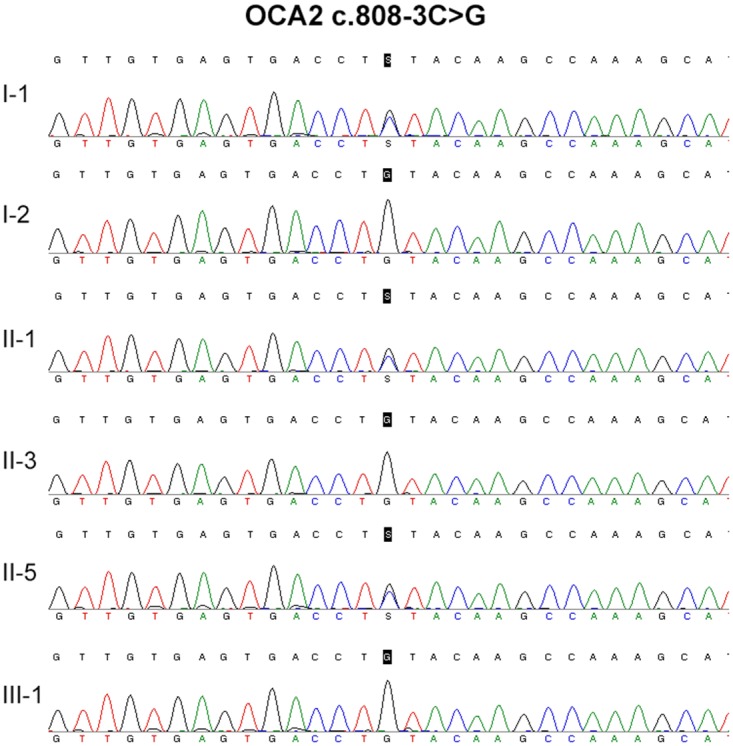
DNA sequencing result of *OCA* c.808-3C>G

**Figure 3:**
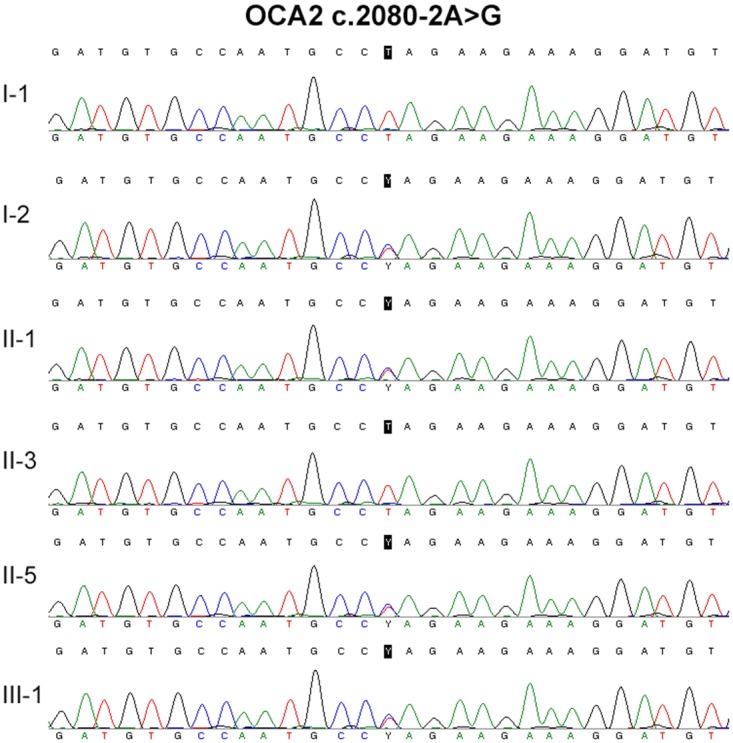
DNA sequencing result of *OCA* c.2080-2A>G

No *TYR* or *TYRP1* mutation was identified in the two patients. Compound heterozygous mutations in *OCA2* (c.808-3C>G and c.2080-2A>G) were identified in the two patients (Figure 2 and Figure 3, Table 1). *OCA2* c.808-3C>G has been previously reported in a Hispanic family with OCA, and this mutation is predicted to affect splicing [10]. *OCA2* c.2080-2A>G mutation is novel, and it has not been found in 1000Genomes (http://www.internationalgenome.org/), Human Gene Mutation Database (HGMD, http://www.hgmd.cf.ac.uk/), or the Exome Aggregation Consortium (ExAC, http://exac.broadinstitute.org/) database.

**Table 1:** Mutation summary of this family member

	OCA2 c.808-3C>G	OCA2 c.2080-2A>G	SLC45A2 c.814G>A	SLC45A2 c.890C>T
I-1	Het	WT	WT	Het
I-2	WT	Het	Het	WT
II-1	Het	Het	WT	Het
II-3	WT	WT	WT	Het
II-5	Het	Het	Het	Het
II-6	WT	WT	WT	WT
III-1	WT	Het	Het	Het

WT, wild type; Het, Heterozygous; Hom, Homozygous.

**Figure 4:**
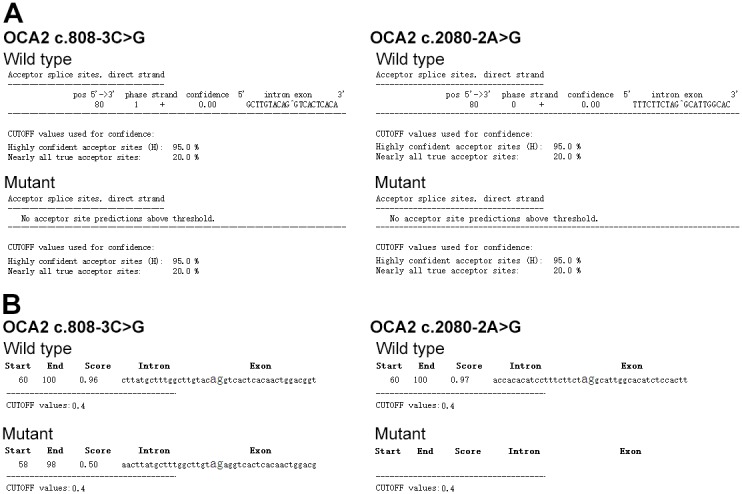
Splice site prediction (**A)**, NetGene2 was used for splice site prediction. (**B)**, NNSPLICE was used for splice site prediction. OCA2 c.808-3C>G wild type (acctagaccgagcagtgccagatcccagatggtgtctcaggtgaaaagcctcaccataacttatgctttggcttgtaCaggtcactcacaactggacggtgt atttaaatccgaggagaagcgagcactcagtgatgagcaggacctttgaggtactgaccaggtgagttctcagtgagtgaggtgttggggcaggctct) and the mutant (acctagaccgagcagtgccagatcccagatggtgtctcaggtgaaaagcctcaccataacttatgctttggcttgtaGaggtcactcacaactggacggtgtatttaaatccgaggagaagcgagcact cagtgatgagcaggacctttgaggtactgaccaggtgagttctcagtgagtgaggtgttggggcaggctct) were used for sequence input. OCA2 c.2080-2A>G wild type (tcattttcaagactttttttttaaatcttgcatatattttcggttctaaactgattctcaccacacatcctttcttctAggcattggcacatctccacttaatagaatatgttggagaacaaactgctttgctaataaaggtaaaataaatgctata atagaaggcactccagccactgttctttgattttgtgaaaaaa) and the mutant (tcattttcaagactttttttttaaatcttgcatatattttcggttctaaactg attctcaccacacatcctttcttctGggcattggcacatctccacttaatagaatatgttggagaacaaactgctttgctaataaaggtaaaataaatgctataatagaaggcactccagccactgttctttgattttgtg aaaaaa) were used for sequence input. The capital words indicate the mutation site.

Several *in silico* analysis programs were used to predict the effect of the compound heterozygous mutations in *OCA2* (c.808-3C>G and c.2080-2A>G) on splicing. The GENIE program performs splice site score calculation. The results indicated that the mutants showed a remarkable decrease of the splice site score (Table 2). The NetGene2 program showed that both *OCA2* c.808-3C>G and c.2080-2A>G mutations abolished a previously predicted splice site (Figure 4A). The NNSPLICE program showed that *OCA* c.808-3C>G mutation generated a novel splice site, and the two nucleotides (AG) from the authentic splice site were incorporated into the coding region, creating a frame shift mutation. The NNSPLICE program showed results similar to those obtained with NetGene2 for *OCA2* c.2080-2A>G (Figure 4B). These data suggest that both *OCA2* c.808-3C>G and c.2080-2A>G mutations may affect *OCA2* mRNA splicing, and compound heterozygous mutations (c.808-3C>G and c.2080-2A>G) in *OCA2* might be responsible for some clinical manifestations of OCA.

**Table 2:** The splice site score for the 3' site calculated by GENIE program

	OCA2 c.808-3C>G	OCA2 c.2080-2A>G
Wild type score	5.2	11.5
Mutant score	-5.3	0.6

**Figure 5:**
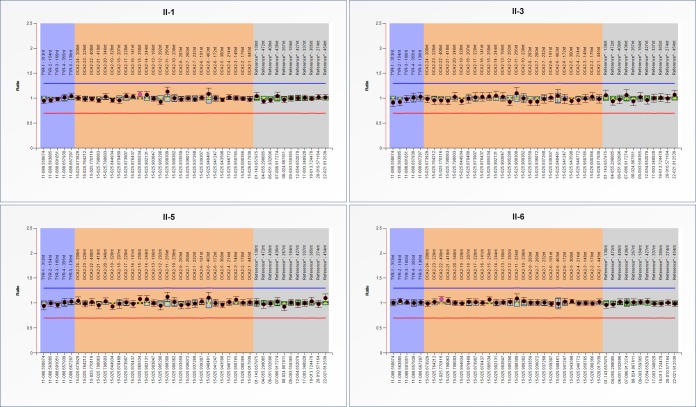
MLPA analysis of TYR and *OCA2* The two patients, their unaffected sister, and the proband’s wife were analyzed for exon rearrangements (deletion/duplication) of *TYR* and *OCA2*.

In order to detect rearrangements (deletion/duplication) of *TYR* and *OCA2*, Multiplex Ligation-dependent Probe Amplification (MLPA) assay was used and the results showed that no significant exon rearrangement (deletion/duplication) of *TYR* and *OCA2* occurred in the two patients (Figure 5).

### Mutation identification and analysis of *SLC45A2*

Compound heterozygous mutations in *SLC45A2* (c.814G>A and c.890C>T, which result in p.Glu272Lys and p.Thr297Ile, respectively) have been identified in both the male patient and a healthy unaffected girl in the family. These data suggest that compound heterozygous mutations in *SLC45A2* (c.814G>A and c.890C>T) may not be associated with OCA in this family.

## DISCUSSION

OCA2 accounts for 30% of all OCA cases worldwide with an estimated prevalence of 1/38,000–1/40,000. It is the most frequent form of OCA in the African population with a higher prevalence of 1/3,900–1/1,500 and in the African-American population, the prevalence is estimated to be 1/10,000 [11, 12]. OCA2 is characterized by variable hypopigmentation of the skin and hair, which may range from minimal to near normal, accompanied with ocular changes. Nystagmus is present before 3–4 months of age. Strabismus and visual inattention may occur in the first six months of age. Iris color ranges from blue to brown. Hair color may darken over time, although the hair color ranges from light yellow to light brown in newborns [13, 14].

OCA2 is caused by mutations in *OCA2*, a human homologue of mouse pink-eye dilution gene located on chromosome 15q11.2-q12 containing 24 exons (23 coding domains). The encoded protein, known as the P protein, is an integral membrane protein composed of 838 amino acid residues that consists of 12 transmembrane spanning regions and is involved in tyrosine transport, which is a precursor to melanin synthesis and pigmentation in the skin, hair, and eye [15, 16]. P protein is involved in the regulation of the pH of melanosomes or served as a melanosomal tyrosine transporter [17]. The most common mutation in *OCA2* is a 2.7-kb deletion, which removes exon 7 and results in a frame shift mutation in the first luminal loop of OCA2 protein, producing a truncated and non-functional protein. This mutation is detected in Africans, sub-Saharan African heritage, African-Americans, and Haitian, suggesting a founder effect [18, 19]. Rooryck *et al.* stated that rearrangements of *OCA2* might be present in more than 20% of patients with OCA2 [20]. P protein may disturb the pigmentation characteristics by altering the melanosomal tyrosine or tyrosinase function due to *OCA2* mutations, but melanocytes of patients with OCA2 still produce small amounts of melanin. OCA2 is caused by homozygous or compound heterozygous *OCA2* mutation, and recessive compound heterozygous mutation indicated that the mutant alleles of both copy are at different locations on the same gene. Patients in the compound heterozygous state may present with a less severe phenotype compared with those presenting with the homozygous form [13]. In the HGMD database, 154 mutations were included, and missense mutations account for 60%, while splicing mutations account for 11%.

No *TYR* or *TYRP1* mutation was identified in the two patients. Two splice site mutations in *OCA2* (c.808-3C>G and c.2080-2A>G) have been identified in both patients, while the healthy family members presented with only one of the two mutations. *OCA2* c.808-3C>G was first identified in a Hispanic family with OCA, and this is the first report in Chinese population. *OCA2* c.2080-2A>G has not been reported in any ethnic population yet. GENIE, NNSPLICE, and NetGene2 programs have been used to predict the effect of the two splice site mutations on *OCA2* mRNA splicing. All three programs showed that both *OCA2* c.808-3C>G and c.2080-2A>G mutations may affect *OCA2* mRNA splicing by abolishing previous splice sites or generating a new splice site. The expression of *OCA2* is very low in leukocytes, and we failed to amplify *OCA2* mRNA. *In vitro* experiments may further confirm these effects.

To exclude the large deletion or duplication of exons in *TYR* and *OCA2*, MLPA was also performed to detect exon rearrangements, and the results showed that no deletion or duplication of exons was found in the patients.

In the current study, compound heterozygous mutations in *SLC45A2* (c.814G>A and c.890C>T) were identified in both a male patient and an unaffected girl in the family. Therefore, compound heterozygous mutations in *SLC45A2* may not be the causative mutation for OCA in this family.

In conclusion, this study expands the mutation spectrum of OCA. Compound heterozygous mutations (c.808-3C>G and c.2080-2A>G) in *OCA2* might be responsible for some clinical manifestations of OCA.

## MATERIALS AND METHODS

### Subjects and clinical evaluation

This study was approved by the Ethics Committee of Tongji Hospital, Tongji Medical College, Huazhong University of Science and Technology. All procedures were carried out in accordance with the approved guidelines. One patient was a 25-year-old male, and another patient was a 37-year-old female. Both of them presented the same clinical ophthalmologic characteristics, including heterochromia iridis, milky skin, yellow hair, photophobia, nystagmus, and reduced visual acuity. Family history and pedigree chart were drawn to evaluate the inheritance model. Written informed consent was obtained from all participants and authorization to publish personal photographs was obtained from the male patient only. However, he only allowed us to publish photographs of his hair and eyebrows, but not of his eyes.

### Strategy for mutational screening

Mutational screening of *TYR* was prioritized for patients with OCA. *OCA2*, *SLC45A2*, and *TYRP1* were sequentially screened for mutations when no mutation was found in *TYR*.

### DNA extraction and mutational analysis

DNA extraction and PCR-based Sanger sequencing were performed as previously described [21]. Briefly, each 50-μL PCR mixture contained 100 ng of genomic DNA, 2 μL of 10 μM forward and reverse primers (with a final concentration of 400 nM), and 25 μL of 2× Taq PCR MasterMix (Takara, Dalian, China). PCR was carried out in Veriti thermocycler (Applied Biosystems, Foster City, CA, USA) using the following protocol: 95°C for 3 min; 35 cycles of denaturation at 95°C for 30 s, annealing at 55°C for 30 s, and extension at 72°C for 45 s; and a final extension at 72°C for 7 min. The amplified products were purified with a cycle-pure kit (Axygen, Wujiang, China) and sequenced using an ABI 3500 Dx sequencer (Applied Biosystems). In order to detect exon rearrangements (deletion/duplication) of *TYR* and *OCA2* and to increase the mutation rate, MLPA assay Kit (P325-OCA2) from MRC-Holland (Amsterdam, Netherlands) was used and the procedure was performed according to manufacturer’s instructions. The mutation was named according to the recommendation of sequence variants by Human Genomic Variation Society (HGVS: http://www.hgvs.org/). The interpretation of sequence variants was made according to the recommendation of the American College of Medical Genetics (ACMG) and Genomics and the Association for Molecular Pathology (AMP) [22]. All primer sequences are listed in Table 3.

**Table 3:** Primer sequences used in this work

Primer name	Sequence	PCR product
TYR CD1 AF	GCT GGA GGT GGG AGT GGT ATT	459bp
TYR CD1 AR	GTC CCC AAA AGC CAA ACT TG	
TYR CD1 BF	AAT GCA CCA CTT GGG CCT C	536bp
TYR CD1 BR	TCC CGC CAG TCC CAA TAT G	
TYR CD1 CF	CAA CAC CCA TGT TTA ACG ACA	475bp
TYR CD1 CR	CAT TGA GAG TTC TTA ACA GGG C	
TYR CD2 F	GAT TTC TCA GAA CAT ATC CCT G	526bp
TYR CD2 R	AGC TAG GGT CAT TGT CGA TAT	
TYR CD3 F	AGA GTC TCA ATA CGG AAT GAA TT	519bp
TYR CD3 R	GTA TCC TGC CTA ATC CAC CTT	
TYR CD4 F	CTG TTT CCA ATT TAG TTT TAT AC	790bp
TYR CD4 R	TAC AAA ATG GCC TAT GTT AAG C	
TYR CD5 F	TGT CTA CTC CAA AGG ACT GT	924bp
TYR CD5 R	GGC ACT TAG CTG GAT GTG TT	
TYR CD4 Sequencing F	CTC CAG ATT TTA ATA TAT GCC	348bp
TYR CD4 Sequencing R	GTG TTA TCT CAA AAT AAA TTG G	
TYR CD5 Sequencing F	GAT GGT GAT CGT AAC AAT GG	311bp
TYR CD5 Sequencing R	TTT GGC CCT ACT CTA TTG CC	
OCA2 CD1 F	CGA AGA AGC AAC CTT CCT ATT GTA C	490bp
OCA2 CD1 R	CTA AGC CAG GAA AGT GAT CTA ATG C	
OCA2 CD2 F	ATT CTT GAA TCT AGC ACC TGA GTG C	306bp
OCA2 CD2 R	TGT CAA GGA TCT GGC AGA GGT TA	
OCA2 CD3 F	ACC CAT TCC CAC CAG TAT GAG AGT	456bp
OCA2 CD3 R	CAA AAC TCA TCC TCT TCT TCA CGC	
OCA2 CD4 F	TGA GAT GGA AGT TAC TCA AGG CTG	285bp
OCA2 CD4 R	AGA CAG TCA GAG AAT CAG GCG AAG	
OCA2 CD5 F	AGT AGC CCC ATC ATC ACA TCT GTT	298bp
OCA2 CD5 R	AAA TTC GAG TGG TAA TGG CCT GT	
OCA2 CD6 F	TTC TTC ACA CAC TGT CAG AGG AGG	382bp
OCA2 CD6 R	GAA TTG ACT AAG AAT GGT GTC CTC G	
OCA2 CD7 F	AAC AAA TAC CTA GAC CGA GCA GTG	242bp
OCA2 CD7 R	TAT AGG TCA GAC TCC TTT AAA CGC A	
OCA2 CD8 F	GCT GTG AGA TTG GGC GTT GG	461bp
OCA2 CD8 R	GCA AAT ATT CCT GTA TGG TTC CCT T	
OCA2 CD9 F	GCC TGA AAC ATC AAG ACC CAT	460bp
OCA2 CD9 R	CCT TTC CTC CAC CAC GAT G	
OCA2 CD10 F	CAG CGA TAT AAT CCA ACT TCA AAG G	355bp
OCA2 CD10 R	GCA CTA ACA CTT CTC AGT CAA GCC	
OCA2 CD11 F	TGT AAG GGA TCA TGC TGA TGT CG	387bp
OCA2 CD11 R	CAC AAC GAT TCA ACC TGA GTA CCC	
OCA2 CD12 F	AAT GTT AGT TTG GCT CCC TGT TCT T	330bp
OCA2 CD12 R	TCA TGC ACC TGA GAA TGG AAC C	
OCA2 CD13 F	ACT CTG GAA AGG AAT GTA ACT CTC G	491bp
OCA2 CD13 R	CTT GAG ATG CCC AGT AGC ACT TAC	
OCA2 CD14 F	ATC CAC CCA CCT CGG AAA GT	329bp
OCA2 CD14 R	AGC ATC CAG CAA CCC ATC AA	
OCA2 CD15 F	GTC TCG AGT GTG TGT CTG CTC TGT C	425bp
OCA2 CD15 R	TGC AGA GCT CAG TGA GGG TTA GAT A	
OCA2 CD16 F	ACA CTC CTT TCA TCA TTC AGG TCA T	423bp
OCA2 CD16 R	AAC CTC AAC GTC TTG TGT ATA ACC A	
OCA2 CD17 F	CTG TCG TGA TTC CAG TTG CGT AG	489bp
OCA2 CD17 R	CAG TGC CCA CTC TAT ATT CCT CCT C	
OCA2 CD18 F	GAG GTA CAA GAA CAT AGG CAT GAA T	552bp
OCA2 CD18 R	AAA TCT CTC AGT GGC TAA GGT AAA G	
OCA2 CD19 F	TCT GGG CCT ACC TTA TGT TCA CG	324bp
OCA2 CD19 R	CAT CTC TGG GCT GCA CAG GAT AG	
OCA2 CD20 F	CTA TGT CTG CCT TGG TCT CGT GAT	379bp
OCA2 CD20 R	CTC TGC TCA CTT TCG TCC TCT ACA C	
OCA2 CD21 F	GGT TTC TTT CCA CAA ATC TTA TGC T	341bp
OCA2 CD21 R	CAT CCA GAC TCT CCT TCA TTT GCT	
OCA2 CD22 F	CAA ATC AAA GCC TGT GAG ATG ATC T	326bp
OCA2 CD22 R	CTC CCC TAC ACC ACA GTC TCT CTA C	
OCA2 CD23 F	GAT GAA CAA ACA GAG GCT CCA	477bp
OCA2 CD23 R	TAG CAT CTC CAG GGT AAG CAC	
SLC45A2 CD1 F	CTG ACC ATC TCT GTT GGT TGC TC	594bp
SLC45A2 CD1 R	CTA GGA AAG GTC AAA CAC ATG AAC A	
SLC45A2 CD2 F	GGA AGA TGA TTT TAT GGC AAG AAG T	357bp
SLC45A2 CD2 R	CGT GTA GAG ACA CTG GAT GGC TT	
SLC45A2 CD3 F	CCC ACT GAA GGG GAG TGT CTA TG	518bp
SLC45A2 CD3 R	CCA TGA AAC TCT TCT CGT CAA ACA G	
SLC45A2 CD4 F	ACA CTT TGT GTG ATG GCT GAC TGA C	358bp
SLC45A2 CD4 R	ACT GTG CCA ATC TTA GAG GAT AGC C	
SLC45A2 CD5 F	GAC ATT TGC TCC CCA GAG GT	451bp
SLC45A2 CD5 R	ACC CAC TGA TTC CAA GAG CAA A	
SLC45A2 CD6 F	CCA CAG ATA AGG GGA TTC TTT TGT T	449bp
SLC45A2 CD6 R	TTC CAG CTC TGC TCT ACA CAT TGC	
SLC45A2 CD7 F	ATC CAC GAA GCC AAA GGT A	459bp
SLC45A2 CD7 R	GAA ATC ACA ATA GTG GGC GT	
TYRP1 CD1 F	TTG AAA GTG GTT TGG GAA GG	742bp
TYRP1 CD1 R	AGC TTC AAC TCC AAC CCT TTA C	
TYRP1 CD2 F	AAT CAT GCA GTA AAT TGG AGA G	687bp
TYRP1 CD2 R	GAA ATG CCA AAG ACA GGT TAG	
TYRP1 CD3 F	CCC TCA GAC ACC GTT GAT ATA CT	499bp
TYRP1 CD3 R	GGT GTT TAA TGA ATG CCT GGT AC	
TYRP1 CD4 F	AAT GGG ACA TGG TAA CTT AGA	570bp
TYRP1 CD4 R	GAT TTC CAA GGG CTT CAC	
TYRP1 CD5 F	GTC ATC AAC CAT AGG TAC AGA G	586bp
TYRP1 CD5 R	GAG AGA TGA TTT GGT TAG TCC	
TYRP1 CD6 F	TAA TTT CTC ATC CTG CTG TAG TG	470bp
TYRP1 CD6 R	ATC AAA TTC CTT CCC TTA TCC	
TYRP1 CD7 F	CTA TCC CAA TAG GGT CCA CTC	558bp
TYRP1 CD7 R	TTC TGA AAG GGT CTT CCC AG	

### *In silico* analysis

To predict the effect of the missense alterations, the following programs were used: InterVar (http://wintervar.wglab.org/), MutationTaster (http://www.mutationtaster.org/), and Ensembl database (http://asia.ensembl.org/index.html) [23, 24]. To predict the splice site, the following programs were used: GENIE program (http://rulai.cshl.edu/new_alt_exon_db2/HTML/score.html), NNSPLICE (http://www.fruitfly.org/seq_tools/splice.html), and NetGene2 (http://www.cbs.dtu.dk/services/NetGene2/) [25–28].
